# TLR-2 expression and dysregulated human Treg/Th17 phenotype in *Aspergillus flavus* infected patients of chronic rhinosinusitis with nasal polyposis

**DOI:** 10.1186/s12934-020-01481-3

**Published:** 2020-11-25

**Authors:** Gargi Rai, Shukla Das, Mohammad Ahmad Ansari, Praveen Kumar Singh, Sajad Ahmad Dar, Shafiul Haque, Neelima Gupta, Sonal Sharma, Vishnampettai Ganapathysubramanian Ramachandran, Sanskriti Sharma, Charu Jain, Shipra Sharma

**Affiliations:** 1 Department of Microbiology, University College of Medical Sciences (University of Delhi) and Guru Teg Bahadur Hospital, Delhi, India; 2 Research and Scientific Studies Unit, College of Nursing and Allied Health Sciences, Jazan University, Jazan, Saudi Arabia; 3 Department of Otorhinolaryngology, University College of Medical Sciences (University of Delhi) and Guru Teg Bahadur Hospital, Delhi, India; 4 Department of Pathology, University College of Medical Sciences (University of Delhi) and Guru Teg Bahadur Hospital, Delhi, India; 5 Sri Venkateswara College, University of Delhi, New Delhi, India; 6 Department of Microbiology, ESIC Medical College, Faridabad, Haryana India; 7 S R Institute of Management and Technology, Lucknow, UP India

**Keywords:** Chronic rhinosinusitis, Nasal polyposis, T helper 17, T regulatory cells, Toll-like receptor, *Aspergillus flavus*

## Abstract

**Background:**

T helper (Th)17 and regulatory T (Treg) cells with toll-like receptor (TLR)-2 have been acknowledged to play a critical role in chronic rhinosinusitis with nasal polyposis (CRSwNP). However, its pathogenesis has been perplexed by conflicting reports on the role of Th17/Treg cells in patients of distinct ethnicities. We attempted to understand the role of Th responses induced during host defense against *Aspergillus flavus.*

**Results:**

The percentages of Th17 (CD4^+^CD161^+^IL23R^+^) and Treg (CD4^+^CD25^+^FoxP3^+^) cell populations and various cytokine profiles in peripheral blood mononuclear cells (PBMCs) challenged by *A. flavus* antigens were characterized from 50 CRSwNP cases, before and after treatment, and in 50 healthy controls. TLR-2 expression was analyzed in tissues of cases and controls for disease co-relation. The major pathogen identified in our study was *A. flavus* by mycological investigations. A marked immune imbalance was noted with elevated Th17 and decreased Tregs in PBMCs of CRSwNP patients after *A. flavus* stimulation. Comparatively, interleukin (IL)-17 and IL-10 levels were increased, with low transforming growth factor (TGF)-β levels in *A. flavus* stimulated PBMC supernatants of patients. The mRNA expression of TLR-2 in polyps of CRSwNP patients indicated significant (*p* = 0.001) upregulation in comparison to the controls.

**Conclusions:**

Our data highlights the excessive expression of TLR-2 in nasal polyps contributing to the imbalance in Th17/Tregs population in patients. After therapy, recovery of Tregs cells indicates restoration and tissue homeostasis, though high circulating CD4^+^CD161^+^ Th17 cells may continue to be a threat to patients predisposed to future recurrences. The constant exposure and tendency of *A. flavus* to colonize nasal cavities can lead to a Th17 driven airway inflammation. Dysregulated Th17 with TLR-2 promote resistance to treatment and progression to the chronicity of the disease.

## Background

Chronic rhinosinusitis with nasal polyps (CRSwNP), a subgroup of chronic rhinosinusitis (CRS), is clinically described by the presence of nasal polyps detectable to the clinician through nasal endoscopy. Nasal congestion, loss of smell, hyposmia and facial pressure are the common clinical presentations [1, 2]. Nasal polyps are characterized by inflammation in the lining of the paranasal sinuses and nasal cavity [3]. The accurate pathogenesis remains unclear, yet different etiologies have been involved, like anatomical variations, microbial disease colonization, fungal infection and atopic reaction [4, 5]. *Aspergillus* species are the most widely recognized colonizers of the sinuses. In India, *Aspergillus flavus* is isolated in more than 80% of the cases of chronic rhinosinusitis [6]. As a pathogen, it cannot effectively damage the mucous membrane as it needs keratolytic proteins. The spores can adhere to particles that are breathed in and stored on the nasal and paranasal sinus mucosa. However, the pathology associated with *Aspergillus* species relies upon the immunologic condition of the patients [7].

It is suggested that there may be a distinctive immune response to fungal antigen in CRS patients that prompts the production of cytokines and drives eosinophilic aggravation. The fungal spore germination in the mucin sends antigenic stimulus, enabling chemo-attraction of various immune cells eventually leading to polyp formation [8–10]. The exact relevance of fungi in CRS still remains unclear, though it is proposed that the pathophysiology of illness is most likely a consequence of mucosal hypersensitivity [11].

Previous reports have shown that T helper (Th) 17 and regulatory T (Treg) cell subsets play unique roles in controlling the inflammatory response. The effector T cell subsets driving immunity and inflammation and the role of inhibitory Treg cells suppressing the effector T cells to limit excessive inflammatory responses are the key components of the immune response [12]. Hence, the balance between immunosuppressive forkhead box P3 (FoxP3) Treg cells and proinflammatory interleukin (IL)-17A secreting cells represents a critical factor in the regulation of immune homeostasis. Strikingly IL-17 has been reported to be involved in atopic irritation in nasal polyps by pulling in the eosinophils and ensuing a tissue response [12, 13]. There is growing proof that toll-like receptors (TLRs) are engaged in modulating Treg cell functions directly and indirectly. Recent studies indicated that TLRs expressed on Tregs are the key component that regulates the immunosuppressive activity. In particular, TLR-2 stimulation appears to decrease the suppressive activity of Tregs by mechanisms that are not completely understood [14–16]. The experimental pathways of Tregs and Th17 cells are considered as divergent and generally inhibitory with IL-17 secretion reportedly being associated with decreased Treg function [14, 17]. Therefore, we hypothesized that TLR-2 may be responsible for reducing the suppressive function of Tregs by regulating the balance between Treg and Th17 function during the course of the disease.

To test this hypothesis, our study aimed to describe the Th responses induced as a host defense against *A. flavus* associated CRSwNP. The TLR-2 expression, Th17/Treg cell markers and profile of various cytokines in peripheral blood mononuclear cells (PBMCs) challenged by *A. flavus* antigens were characterized in CRSwNP cases before (NP) and after treatment (NPF) and compared with healthy controls (HC).

## Results

### Patient profiles

Patients with CRSwNP had presented predominantly with symptoms of nasal obstruction, nasal discharge, irritation and sneezing. Clinical examination included rhinoscopic examination followed by other routine laboratory investigations. Symptom score was assessed for disease severity according to the visual analogue scale (VAS), and the preoperative computed tomography (CT) scores calculated and graded. Overall, patient profile and clinical characteristics are summarized in Table 1. All the patients underwent functional endoscopic sinus surgery (FESS) with a clearance of paranasal sinuses, to the best extent possible. Patients were subsequently followed up for 6 months after surgery and were put on systemic steroids for 2 weeks and intranasal steroids for a prolonged duration. Nasal washes with saline were also recommended in all cases.

**Table 1:** Profile and characteristics of CRSwNP cases and healthy controls

Parameters	CRSwNP	HC
Subjects (n)	50	50
Gender (M/F)	29/21	26/24
Age (years)	27 (16–50)	28 (18–49)
Duration of disease (in months)	11 (4–32)	0
CT score	14 (7–18)	0
VAS score	13 (10–16)	0

Data are expressed as medians and interquartile ranges

All the patients who were positive for *Aspergillus* sp. by KOH, culture and histopathological findings, were considered for this study. A total of 44/50 (88%) and 6/50 (12%) were found to be positive for *A. flavus* and *A. fumigatus*, respectively

### CD3^+^CD4^+^ T cells expressed higher Th17 (CD161^+^ and IL-23R^+^) cells in PBMCs NP patients after* A. flavus* stimulation

**Fig. 1:**
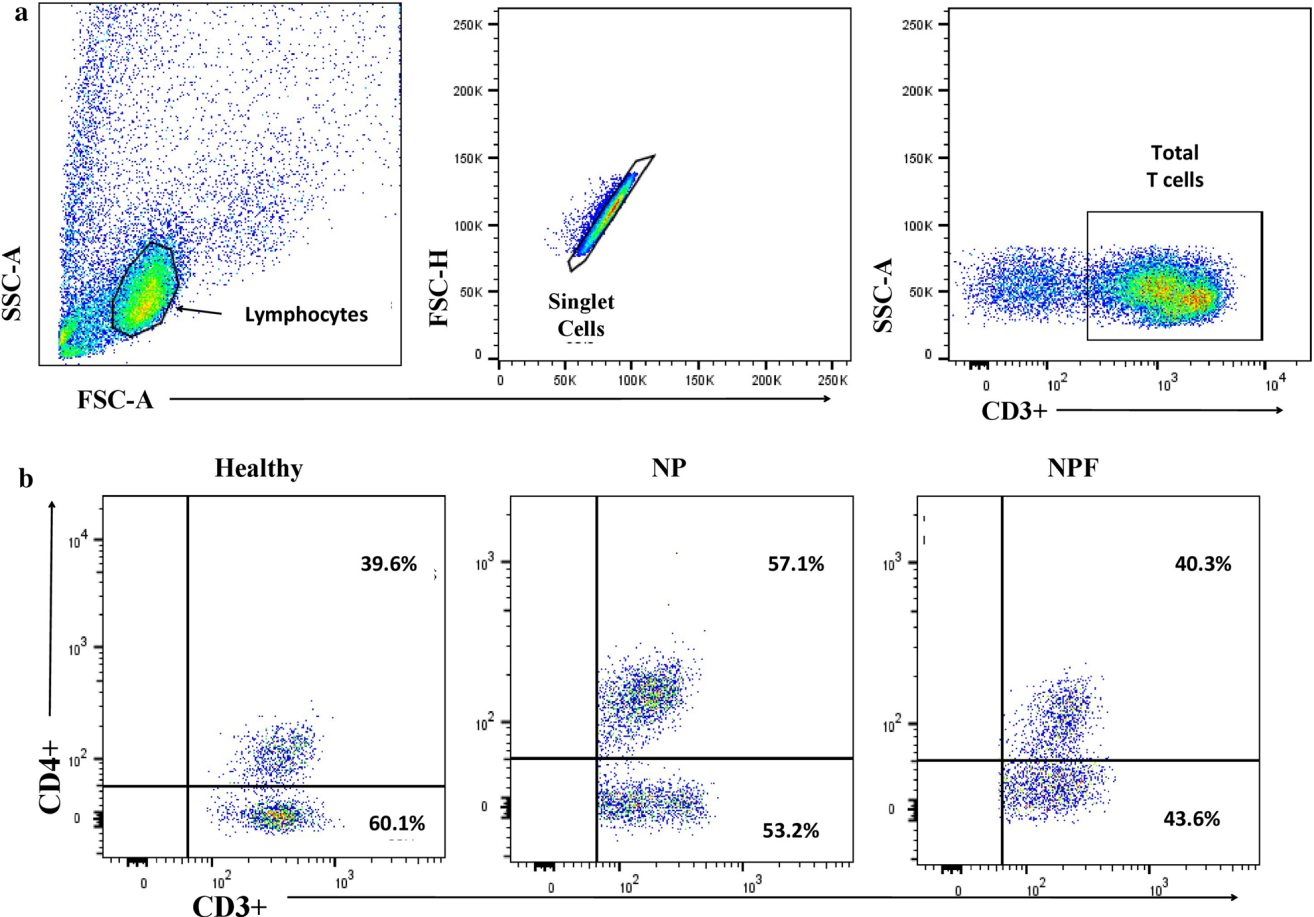
Gating strategy and T cell subset expression. Lymphocytes were analyzed with flow cytometry after stimulation with PHAM and *A. flavus* antigen and in unstimulated cells for 18 h. **a**,** b** Gating strategy for CD3^+^ T cells from lymphocytes and representative FACs plot showing CD4^+^ T cells in the total CD3^+^ T cells, in CRSwNP case before (NP) and after treatment (NPF) and healthy controls (HC)

**Fig. 2:**
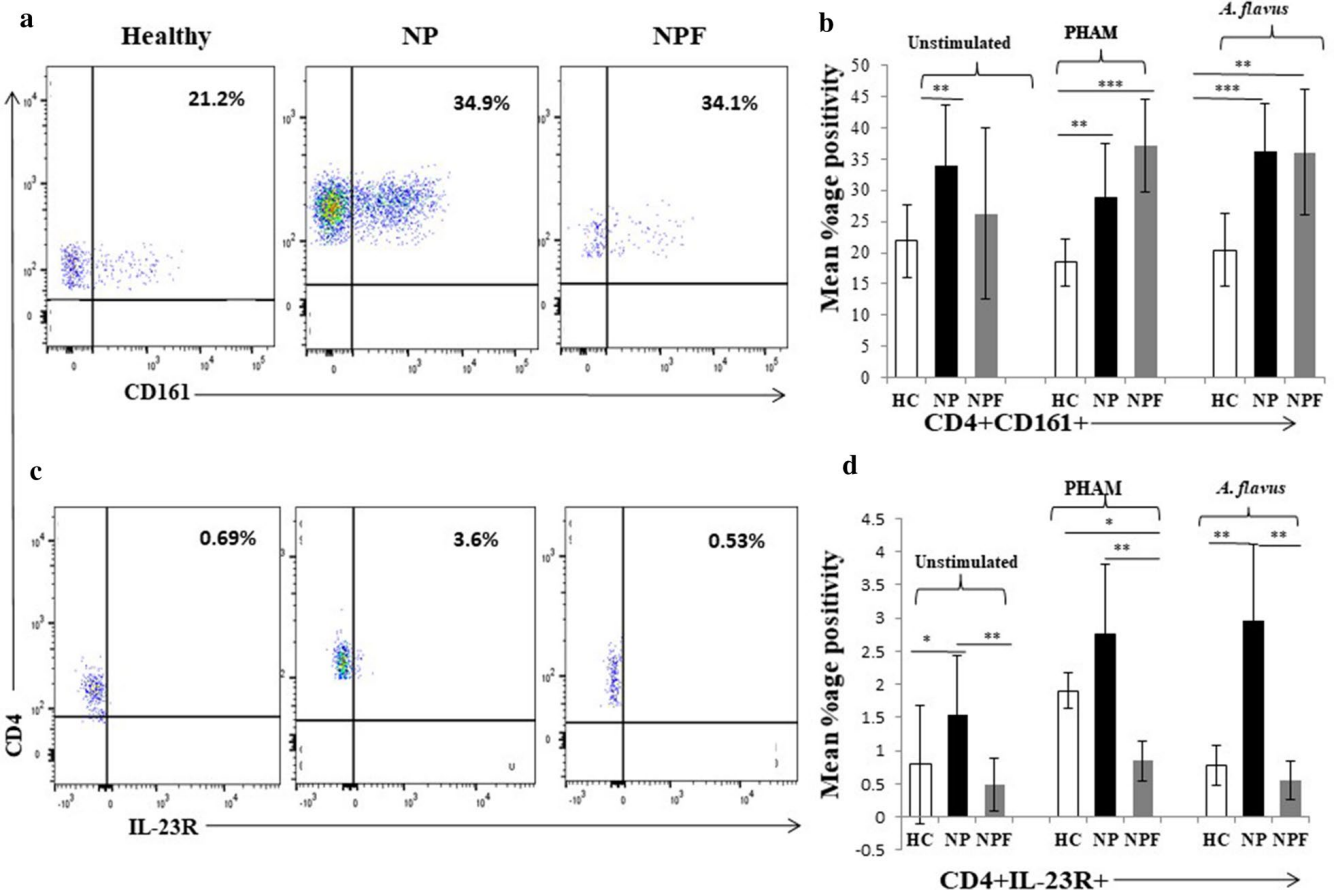
CD3^+^CD4^+^ T cells expressed higher CD161^+^ and IL-23R^+^ Th17 cells percentages in PBMCs of CRSwNP. Lymphocytes were analyzed with flow cytometry after stimulation with PHAM and *A. flavus* antigen and in unstimulated cells for 18 h. **a**, **b** Representative FACS plots showing the percentage positivity of CD161^+^ in CD4^+^ T cells and **c**,** d** representative FACS plots showing the percentage positivity of IL-23R^+^ in CD4^+^ T cells, in CRSwNP cases before (NP) and after treatment (NPF), and healthy controls (HC) after stimulation with PHAM and *A. flavus* antigen and in unstimulated cells The statistical significance was determined by the Student t-test. Where * *p *  ≤ 0.05; ** *p * ≤ 0.01; *** p ≤  0.001

We first analyzed the percentages of CD3^+^CD4^+^ T cells by flow cytometry, and observed higher percentages of CD4^+^ T cells in patients than controls (Fig. 1b). CD4^+^ cells expressing CD161^+^ and IL-23R^+^ (Th17 cells) were found in higher percentages in NP as compared to HC in unstimulated as well as after *A. flavus* and PHAM stimulation (*p* < 0.05, Fig. 2). CD161^+^ and IL-23R^***+***^ Th17 cells were again analyzed in CD4^+^ T cells population after a follow-up of 6 months, where a reduced expression of CD4^+^CD161^+^ cell markers in unstimulated cells in NPF patients as compared to NP and nearly similar to those of the HC was observed. In *A. flavus* stimulated cell population, CD161^+^ markers on CD4^***+***^ T cells were found to be increased in NPF patients as compared to HC, but no difference was found between NPF and NP patients groups. (Fig. 2a, b). CD4^+^IL-23R^+^ Th17 population was found to be significantly increased in NP as compared to NPF and HC groups in unstimulated and *A. flavus* stimulated cells. The percentage of CD4^+^IL-23R^+^ Th17 cells in NPF group showed decreased response as compared to NP though the cell population was comparable to HC in *A. flavus* stimulated cell population (Fig. 2c, d).

### Decreased Tregs (CD25^+^FoxP3^+^) expression on CD3^+^CD4^+^ T cells in NP after the challenge of PBMCs with* A. flavus* antigen

**Fig. 3:**
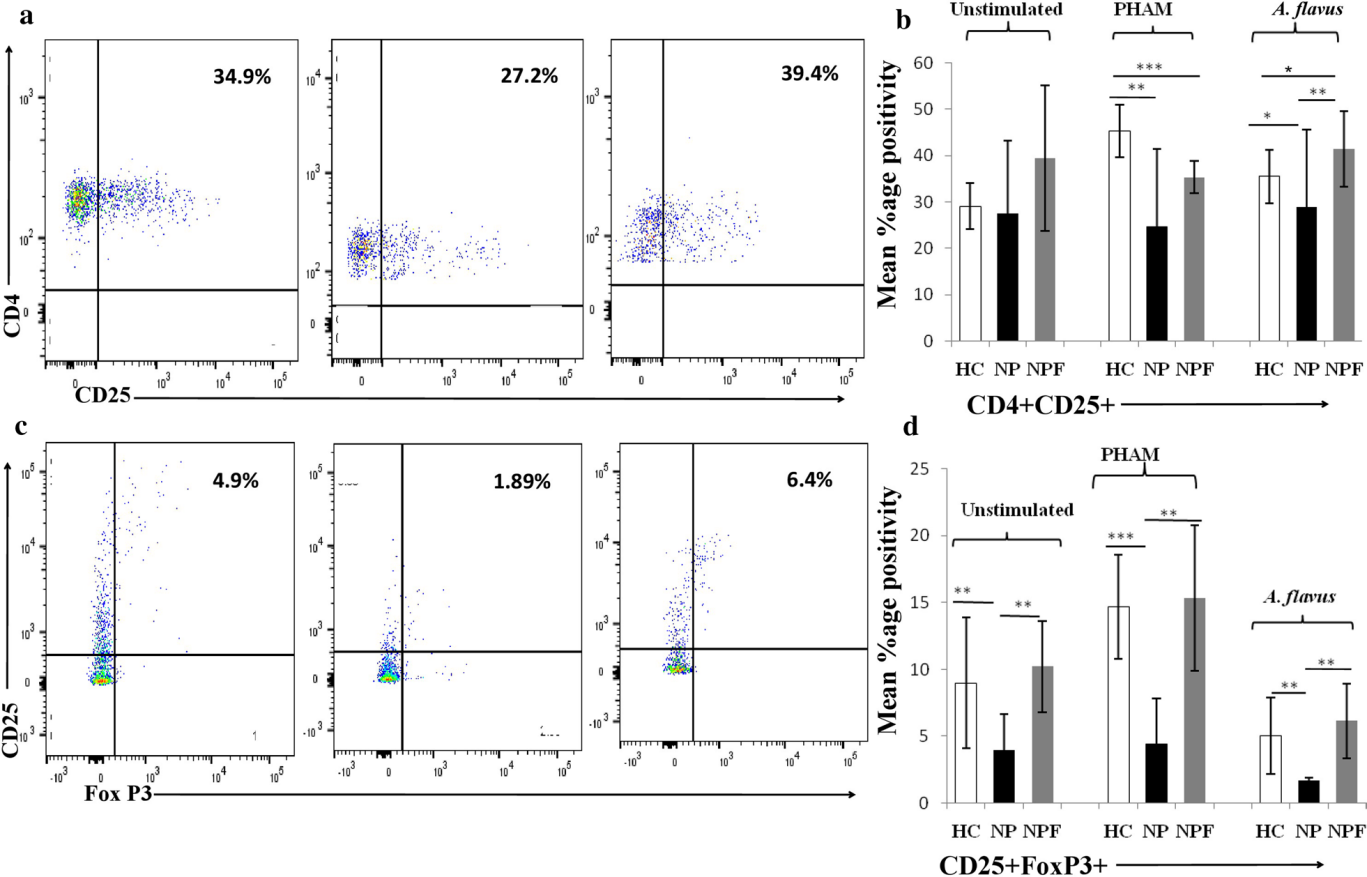
CD3^+^CD4^+^ T cells expressed a decreased percentage of CD25^+^FoxP3^+^ Treg cells in PBMCs of CRSwNP. **a**, **b** Representative FACS data and summary analysis showing the percentage positivity of CD25^+^ in CD4^+^ T cells and **c**, **d** representative FACS data and summary analysis showing the percentage positivity of CD25^+^FoxP3^+^ in CD4^+^ T cells, in CRSwNP case before (NP) and after treatment (NPF) and healthy controls (HC) after stimulation with PHAM and *A. flavus* antigen and in unstimulated cells. The statistical significance was determined by the Student t-test. Where * *p  * ≤ 0.05; **p  ≤ 0.01; ***p  ≤ 0.001

When unstimulated, the CD4^+^CD25^+^ Treg population showed a significant decrease in NP as compared to HC and follow-up NPF groups. In *A. flavus* stimulated cells, CD4^+^CD25^+^ Treg cells showed a significant increase in NPF as compared to HC group (Fig. 3a, b). Dual positive CD4^+^CD25^+^FoxP3^+^ Treg cells showed significantly decreased response in NP as compared to HC and NPF groups in unstimulated, PHAM and *A. flavus* stimulated cell populations. Moreover, CD25^***+***^FoxP3^+^ showed similar results in NPF and HC groups in unstimulated and after stimulation with PHAM and *A. flavus* (Fig. 3c, d).

### Cytokine estimation

**Fig. 4:**
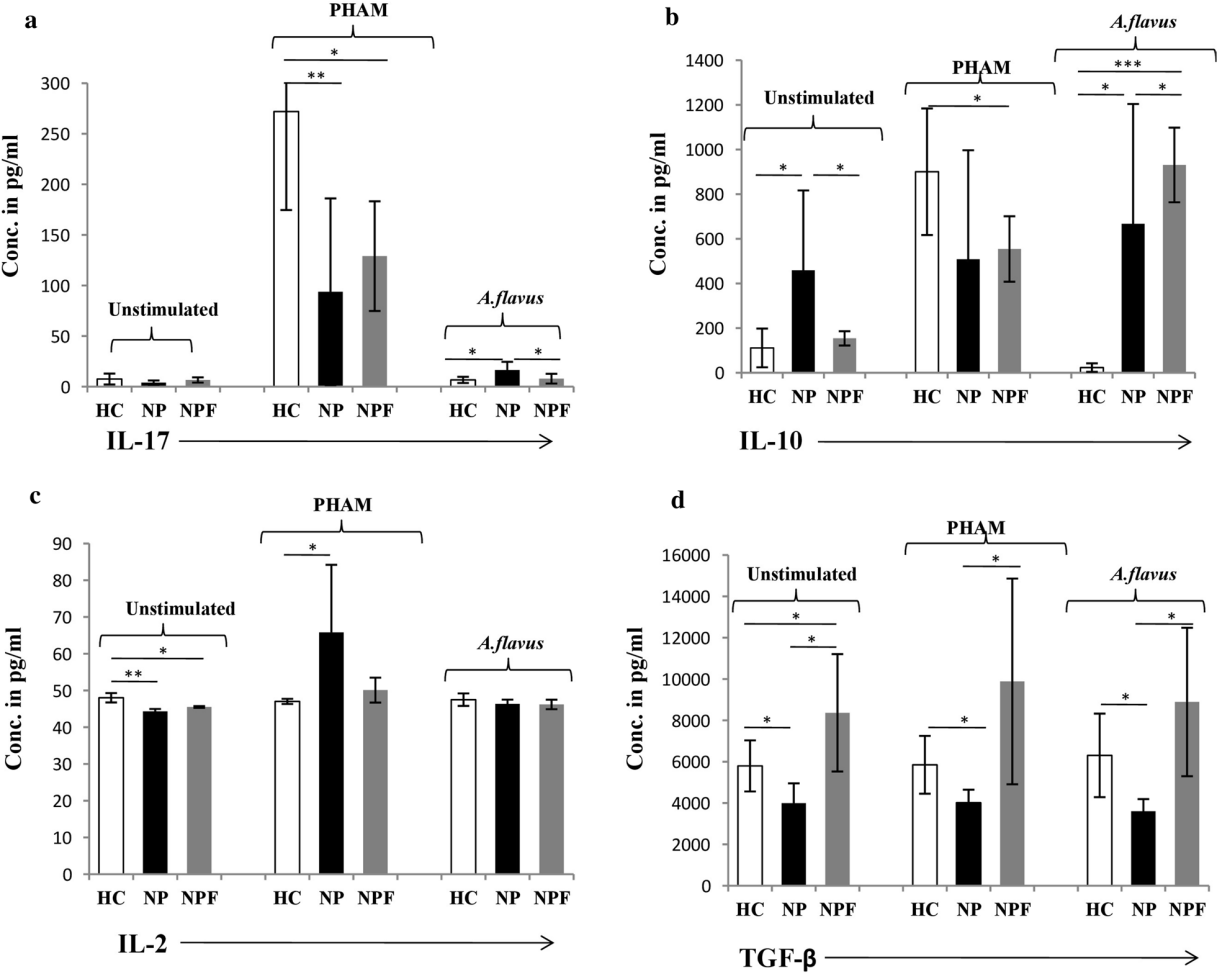
Graphical representation of cytokine (IL-17, IL-10, IL-2 and TGF-β) estimation in CRSwNP case before (NP) and after treatment (NPF) and healthy controls (HC) after stimulation with PHAM and *A. flavus* antigen and in unstimulated cells. Where * *p* ≤  0.05; ** p ≤ 0.01; *** *p* ≤ 0.001

The cytokine analysis showed no significant difference in IL-17 level in the NP group as compared to HC and NPF groups, in the absence of any stimulation. After stimulation with *A. flavus* antigen, the levels of IL-17 were significantly increased in NP as compared to HC and NPF groups (Table 2; Fig. 4a). A high IL-10 level after *A. flavus* stimulation was significant in NPF as compared to both NP and HC groups, though IL-10 levels in NP groups was higher as compared to HC (Table 2; Fig. 4b). The IL-2 levels in unstimulated population in NP were lower as compared to HC. A PHAM treatment showed high IL-2 in NP as compared to HC and NPF groups, whereas no significant difference was found within any groups after *A. flavus* stimulation (Table 2; Fig. 4c). TGF-β levels were significantly decreased in NP as compared to the rest of the groups (HC and NPF) in unstimulated, PHAM and *A. flavus* stimulated populations. Although, TGF-β was higher in NPF compared to HC group (Table 2; Fig. 4d).

**Table 2:** Cytokine profile of CRSwNP cases and healthy controls after stimulation with antigen:

Cytokines	Antigen/stimulant	HC	*p-*value	NP	*p-*value	NPF	*p-*value	HC
IL-17 (pg/ml)	Unstimulated	7.49 ± 5.4	0.224	3.94 ± 2.16	0.07	6.57 ± 2.59	0.74	7.49 ± 5.4
	PHAM	272 ± 97.4	0.01	93.8 ± 92.31	0.39	129 ± 54.23	0.02	272 ± 97.4
	*A. flavus*	6.63 ± 3.06	0.013	16.37 ± 8.12	0.032	7.89 ± 4.83	0.61	6.63 ± 3.06
IL-10 (pg/ml)	Unstimulated	111.07 ± 86.7	0.029	458.8 ± 357.8	0.047	154 ± 32.1	0.26	111.07 ± 86.7
	PHAM	900.43 ± 283.64	0.079	508.25 ± 488.38	0.807	554.25 ± 146.58	0.02	900.43 ± 283.64
	*A. flavus*	23.00 ± 19.17	0.019	667.36 ± 536.4	0.025	930.83 ± 167.21	0.00	23.00 ± 19.17
IL-2 (pg/ml)	Unstimulated	48.02 ± 1.27	0.01	44.3 ± 0.67	0.081	45.5 ± 0.23	0.012	48.02 ± 1.27
	PHAM	47.03 ± 0.70	0.035	65.8 ± 18.39	0.06	50.1 ± 3.39	0.07	47.03 ± 0.70
	*A. flavus*	47.5 ± 1.7	0.23	46.34 ± 1.17	0.93	46.2 ± 1.29	0.23	47.5 ± 1.7
TGF-β (pg/ml)	Unstimulated	5796 ± 1236.64	0.027	3990 ± 964.1	0.012	8365 ± 2840.31	0.039	5796 ± 1236.6
	PHAM	5850 ± 1398.62	0.039	4016.25 ± 628.6	0.034	9885 ± 4975.68	0.107	5850 ± 1398.6
	*A. flavus*	6306 ± 2021	0.039	3603.75 ± 584.7	0.015	8890 ± 3590.87	0.172	6306 ± 2021

Data are expressed as mean ± SD and *p* ≤ 0.05 considered as significant

*HC* healthy controls,* NP* cases before treatment,* NPF* cases after treatment,* pg/ml* picogram/milliliter

### Increased TLR-2 mRNA expression by RT-PCR and its correlation with T cell markers and cytokines

**Fig. 5:**
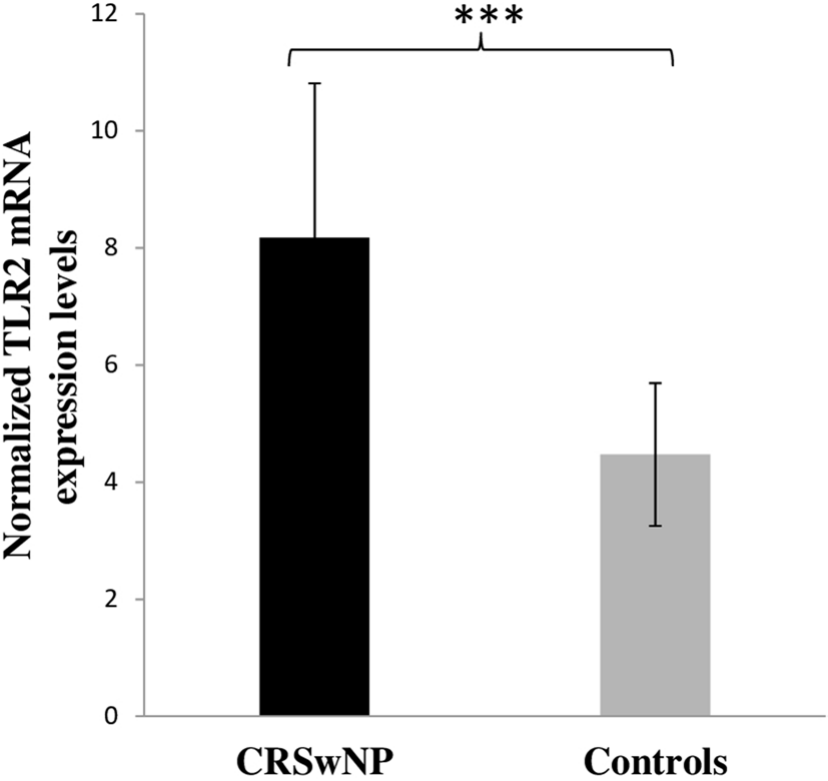
Elevated expression of TLR-2 in patients of CRSwNP as compared to controls. Gene expression was normalized by β-actin. ***p = 0.001

**Fig. 6:**
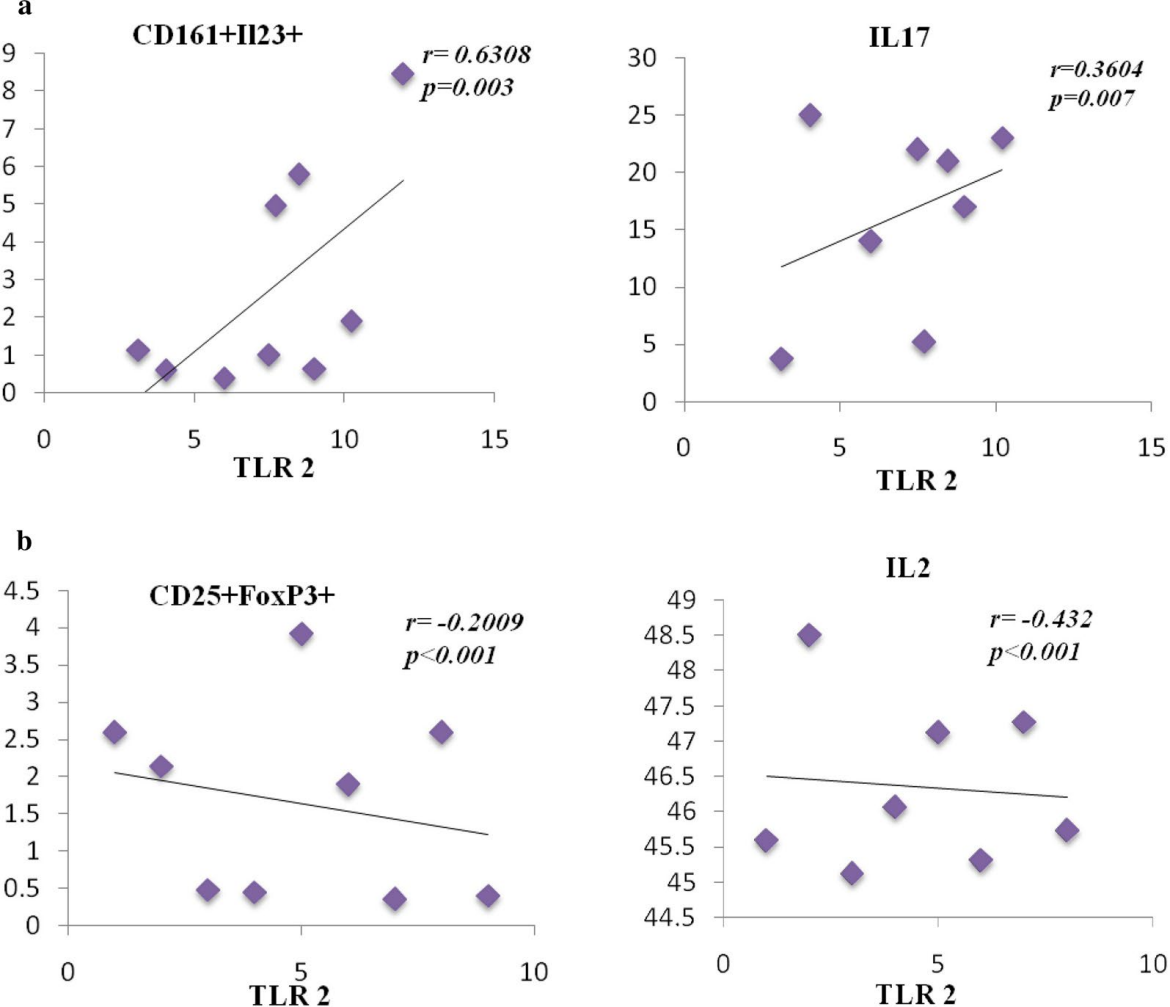
TLR-2 showed a positive correlation with Th17 and negative with Tregs cells. Correlation analysis of mRNA expression of TLR-2 with percentage positivity of T cells markers and cytokine levels in PBMCs supernatants. **a** Correlation between CD161^+^IL-23R^+^ and IL-17 expression for Th17 cells in PBMC of CRSwNP patients, **b** Correlation between CD25^+^ FoxP3^+^ and IL-2 expression for Treg cells in PBMC of CRSwNP patients

The mRNA expression of TLR-2 was remarkably upregulated (*p* = 0.001) with a mean ± SD of 8.18 ± 2.63 in CRSwNP patients as compared to uncinate tissues (controls) 4.47 ± 1.22 (Fig. 5). We examined the correlations between the CD161^+^IL-23R^+^ (Th17) and CD25^+^FoxP3^+^ (Treg) cell populations with TLR-2 expression in CRSwNP patients (*A. flavus* stimulated levels). Also deduced were the correlations between IL-17 and IL-2 with TLR-2 (Fig. 6a, b). Our analysis confirmed a significant positive correlation between TLR-2 expression and CD161^+^IL-23R^+^ cell population (r = 0.6308, *p* = 0.003). We found a significant negative correlation between CD25^+^FoxP3^+^ and TLR-2 expression (r = − 0.2009, *p* < 0.001). Further, expression of TLR-2 showed a significantly positive correlation with IL-17 (r = 0.3604, *p* = 0.007), and a significantly negative correlation with IL-2 (r = − 0.432, *p* < 0.001) (Fig. 6a, b). No significant correlation was observed with IL-10 and TGF-β.

## Discussion

CRSwNPs is a persistent inflammatory condition affecting the nose and paranasal sinuses. Various etiologies, like anatomical variations, fungal infection as well as colonization, and atopic reaction with dysregulated immune response have been described as some of the underlying factors [4, 5]. The current study shows that fungal colonization in CRSwNP patients is associated with defined local immune reactivity, demonstrated a high population of Th17 cells and related cytokines in peripheral blood circulation. Increased percentage of CD161^+^IL-23R^+^ and high IL-17 with a decreased percentage of CD25^+^FoxP3^+^ and low TGF-β were also observed. The high production of IL-17 on in-vitro *A. flavus* stimulation of PBMCs in CRSwNP patients defines a probable modulation of Th17 effector responses during infection. Though IL-17 levels were low in unstimulated population, surprisingly on stimulation with *A. flavus* the levels increased significantly in patients. It’s highly probable that persisting as nasal colonizers in patients, *A. flavus* driven IL-17 production may trigger a chronic granulomatous reaction and polyp formation. Strikingly the low Tregs CD25^+^FoxP3^+^ population accompanied by reduced levels of TGF-β in CRSwNP patients are additional factors playing a detrimental role leading to chronic inflammation. Thus, assuming such an underlying mechanistic dysregulation in Treg cell population, an uncontrolled *A. flavus* driven an increase in Th17 cell population in CRSwNP patients becomes inevitable, contributing to exacerbation of inflammation and disease progression [12, 18]. Moreover, the impaired Tregs cell functionality as a consequence of low TGF-β levels becomes detrimental in these patients and further reduces the chances of remission. On follow-up at 6 months of treatment, we observed a significant increase in TGF-β levels compared to the pre-treatment levels in patients. Meanwhile, IL-2 levels remained unaffected and not much variation was observed even after in-vitro *A. flavus* stimulation in both patients and healthy controls. During recovery, a high TGF-β and co-existing IL-2 cytokine levels may have a beneficial impact in activating the Treg cell population thereby permitting inhibition of Th17 cell differentiation thus lowering inflammation [19–21]. Hence, a complex interplay of Treg/Th17 are the driving essentials in regulating the disease process in CRSwNP patients and further *A. flavus* convincingly modulates the severity and the course of the disease. In contrast, the production of IL-10 was significantly elevated on in-vitro stimulation with *A. flavus*, in patients before treatment and even at 6 months follow up after treatment. Persisting elevated levels of IL-10 in patients with CRSwNP may worsen local allergic activity and adversely affect the effector phagocytes antifungal activity [22–24]. IL-10, an important cytokine produced by Treg cells, was increased in patients with NP in our study which is contrary to the result of Tregs in unstimualted conditions. A recent report describes the independent existence of a T cell subset (Th9), having IL-10 effector secretion [12, 25]. The high IL-10 in the absence of an antigenic stimulus (post FESS and removal of the nasal polyp) may have a beneficial role by suppression of Th17 signals, thus enabling tissue repair, as explained in previous studies [22–24]. While in the absence of an antigenic stimulus (post FESS) it manifests a protective role in the suppression of Th17 signals thus enabling tissue repair. The patterns of TLR expression in CRSwNP patients with fungal colonization compared to healthy controls generates great interest. Recent studies indicate that the upper airway mucosa is an important immunological sentinel location and epithelial cells expressing TLRs have been recognized to play a key role in distinguishing pathogen and regulating the innate and adaptive immune response [26–30]. Also, the fact that mast cells from nasal polyps express a unique profile of TLRs, suggest their specialized role in the local host response to fungal pathogens [31, 32]. Pathogen recognition receptors from the family of TLRs are crucial in generating effective immunity and establish a direct link between Tregs and effector T cell response [33–35]. Moreover, TLR-2 stimulation polarizes IL-10 production causing persistence of infection [28]. We observed the level of TLR-2 expression was remarkably upregulated in CRSwNP patients as compared to uncinate tissue controls. The overexpression of TLR-2 mRNA is essentially markers of excessive inflammation, which may cause exaggerated signal cascade, triggering uncontrolled cytokine formation, contributing to the formation of nasal polyps [31, 35]. TLR-2 expression showed a significant positive correlation with high Th17 cells (CD161^+^IL-23R^+^) and IL-17 while a negative correlation with Tregs cells (CD25^+^FoxP3^+^) and IL-2 and IL-10 levels. According to the previous report, TLR-2 signaling enhances the Th17 immune responses and promotes the development of several fungal diseases [36]. Earlier studies also demonstrated that TLR-2 ligation leads to the decreased suppressive role of Treg cells [16]. In the presence of various inflammatory cytokines, such as IL-6 and IL-1β, Tregs on activation generate IL-17, and an uncontrolled high IL-17 may eventually cause reduction of Treg suppressive role. This remark is associated with the reciprocal and mutually inhibitory modulation of Th17 and Treg cells development [37, 38].

Furthermore, activated CD4^+^ T cells displaying greater TLR-2 expression act as a co-stimulator for antigen-specific T cell development, eventually contributing to T cell memory maintenance [39].

The expression of CD161^+^ on T cell subsets are a hallmark of the Th17 population [40]. In the present study, we found CD161^+^ cell population remained high as compared to healthy controls even after surgery at 6 months follow up. In this respect, it raises serious concern that persistence of residual memory Th17 phenotypes in circulation indicates their ability to trigger future relapses despite complete treatment and surgical management. Conversely, the IL-23R levels remained low after treatment compared to the pre-treatment levels. Additionally, the remarkable recovery of CD4^+^CD25^+^ cells conveyed their plasticity hence subverted Tregs in nasal polyps could once more regain their suppressive role after treatment.

The present findings suggest TLR-2 stimulation as an alternative route for the retention of Th17, causing chronic nasal polyposis, *A. flavus* is capable of inducing Th17 cells detrimental to a human host, and reversal to the anti-inflammatory state by Treg appears to be a promising strategy for therapy.

## Conclusion

The study highlights excessive expression TLR-2 in nasal polyps contributing to the imbalance in Th17/Tregs population in patients of chronic rhinosinusitis. The constant exposure and tendency of *A. flavus* to colonize nasal cavities can lead to a Th17 driven inflammation. Additionally, an IL-10 predominance though induce macrophage activation but remain less efficient in phagocytic activity. Hence, Th17 with TLR-2 promotes resistance to treatment and progresses to chronicity. TLRs are considered to play a role in the pathogenesis of CRSwNP. A possible role of TLR specific treatment can be explored for the future therapeutic regimen.

## Methods

The study was a prospective, analytical, case-control study, done in a tertiary hospital in Delhi, India from April 2016 to December 2018. The study was carried out in accordance with the WHO recommendations, and the approval of the Institutional Ethics Committee—Human Research of the University College of Medical Sciences, University of Delhi, Delhi. The written informed consent was obtained from all subjects before the collection of the samples.

### Patients and samples

Subjects enrolled in the study included fifty CRSwNP patients from the associated hospital undergoing FESS. The diagnosis of nasal polyposis was made according to the definition by the European Position Paper on Chronic Rhinosinusitis and Nasal Polyps criteria (EPOS 2012) and was based on clinical examination, patient history, nasal endoscopy and sinus CT scanning. Fifty healthy volunteers without any history of nasal/sinus surgery were taken as a control group. The VAS was used to assess symptom scores and the CT scan of nose and paranasal sinuses were scored by the Lund and Mackay classification [41]. Preoperatively, blood samples were collected from the patients and healthy volunteers in EDTA vials (6 ml) for PBMCs separation. Post-operatively, polyp biopsy samples were obtained in normal saline and formalin and transferred immediately to the laboratory for examination. Uncinate tissues from patients undergoing septoplasty were taken as controls for biopsies. Tissue biopsies were subjected to direct KOH (10%) examination and culture on Sabouraud Dextrose Agar with antibiotics (0.4 g/l chloramphenicol, 0.04 g/l gentamicin) and incubated at 25 °C. The rate of growth, surface texture and pigmentation were noted. Standard Tease Mount using Lacto Phenol Cotton Blue was prepared from the growth in culture for identification of *A. flavus*. The tissue samples in 10% formalin were subjected to histopathological examination using hematoxylin and Eosin/Gomori Methenamine Silver staining for demonstrating fungal hyphae, eosinophils, neutrophils, Charcot Layden Crystals, inflammatory cells and evidence of tissue invasion. Tissue biopsy from CRSwNP cases and controls were stored in liquid nitrogen for further RNA extraction. The procedures and assays were performed at the time of enrollment of patients and repeat blood samples were collected from each patient for isolation of PBMCs 6 months later after specific therapy prescribed by concerned Otorhinolaryngologists.

### PBMCs isolation and cell culture

From the whole blood samples, PBMCs were isolated using Ficoll-Hypaque density gradient centrifugation method. The viability of the cells was measured by a trypan blue dead cell exclusion assay. Cells were re-suspended in RPMI 1640 media (containing 100 U/ml penicillin, 100 mg/ml streptomycin, 2 mM glutamine and 10% heat-inactivated fetal calf serum) at a concentration of 1 × 10^6^ cells/ml. PBMCs were plated in 48 wells culture plate and were treated with phytohemagglutinin-M (PHAM) mitogen at 10 µg/ml (Hi-Media Laboratories Pvt. Ltd., India) and *A. flavus* antigen 20 µg/ml (All Cure Pharma, Delhi, India) for 18 h at 37 °C in a 5% CO_2_ atmosphere. The cells were harvested and centrifuged for 5 min at 1200 rpm. The cells were collected and stained for surface, and intracellular markers for flow cytometry whereas cell-free supernatants were assayed by enzyme-linked immunosorbent assays (ELISA) for cytokine estimation. The supernatants were stored at − 80 °C till tested for the cytokines.

### Flow cytometry staining

The cells were harvested and washed twice with phosphate-buffered saline buffer containing 0.1% bovine serum albumin and 0.05% sodium azide. The expression of surface and intracellular markers was analysed by immunostaining PBMCs with the antibodies against CD3, CD4, CD25, FoxP3, CD161, IL-23R; (BD PharMingen, USA). For surface staining, cells were incubated with the relevant antibodies at 4 °C in the dark for 30 min. For detection of intracellular FoxP3, CD4CD25 cells were fixed with fixation and permeabilization buffer (BD Bioscience PharMingen, USA) and stained according to permeabilization/fixation Kit protocol. The doublets were excluded; by opting height versus width FSC gating in all the flow cytometry experiments before sample acquisition (Fig. 1a). Suitable fluorescence minus one (FMO) controls was used to define the negative population in all the experiments. The stained cells were then analyzed by flow cytometry (FACS, ARIA III, BD Biosciences, USA). Fluorescence profiles were analyzed using FlowJo software (BD Biosciences). The results expressed as a percentage of positive cells.

### ELISA

The cytokines IL-17, IL-10, IL-2, and TGF-β were determined using commercially available ELISA kits (Diaclone, France) following the manufacturer’s instructions. The detection limits were, 2.3 pg/ml for IL-17, 4.9 pg/ml for IL-10, 7 pg/ml for IL-2 and 8.6 pg/ml for TGF-β. All values below the detectable limit were considered to be zero for easy assessment.

### Real-time PCR analysis for TLR-2 mRNA expression

RNA was extracted from nasal polyp tissue from cases and uncinate tissues for control using TRIzol™ Reagent (Invitrogen, USA) as per the manufacturer’s instructions and was reverse-transcribed to cDNA with random hexamer primers and RNAase H-reverse transcriptase (Invitrogen, USA). Expression of mRNA was determined on the LightCycler^®^ 480 Instrument (Roche, Germany) using SYBR Green Master Mix (Roche, Germany). The following primers were used for TLR-2: forward 5′-GGCCAGCAAATTACCTGTGTG-3′, reverse 5′-CCAGGTAGGTCTTGGTGTTCA-3′; β-actin: forward 5′- AAGATGACCCAGATCATGTTT GAGACC-3′, reverse 5′-AGCCAGGTCCAGACGCAGGAT-3′ [42]. All PCRs were performed in duplicate. Relative gene expression was calculated using the comparative CT method. β-Actin was used as a housekeeping gene for normalization, and a no template sample was used as a negative control.

### Statistical analysis

Data analysis was done using SPSS (SPSS Inc., Chicago, USA; version 20.0). Comparisons between two groups were performed with the independent student t-test. Pearson’s coefficient correlation (r) test was performed to determine correlations between TLRs and T cell subpopulations. All the tests were two-tailed with the significance level at probability below 0.05.

## Data Availability

All data generated or analyzed during this study are included in this published article.

## Abbreviations

*A. flavus*: *Aspergillus flavus*
CRS: Chronic rhinosinusitis
CRSwNP: Chronic rhinosinusitis with nasal polyposis
CT: Computed tomography
EPOS: European Position Paper on Rhinosinusitis and Nasal Polyps
ELISA: Enzyme-linked immunosorbent assay
FESS: Functional endoscopic sinus surgery
FMO: Fluorescence minus one
FoxP3: Forkhead box P3
HC: Healthy controls
IL: Interleukin
NP: Chronic rhinosinusitis with nasal polyposis cases before treatment
NPF: Chronic rhinosinusitis with nasal polyposis cases after treatment
PBMCs: Peripheral blood mononuclear cells
PHA-M: Phytohemagglutinin-M
SD: Standard deviation
*sp*: Species
TGF: Transforming growth factor
Th: T-helper
TLR: Toll-like receptor
Treg: T-regulatory
VAS: Visual analog scale
